# The Human in the Middle: Artificial Intelligence in Health Care Summary Proceedings Symposium Presentation and Reactor Panel of Experts Thomas Jefferson University December 10, 2019

**DOI:** 10.1089/pop.2020.0030

**Published:** 2021-04-09

**Authors:** Janice Clarke, Alexandria Skoufalos, Stephen K. Klasko

**Affiliations:** ^1^ Jefferson College of Population Health of Thomas Jefferson University, Philadelphia, Pennsylvania, USA.; ^2^ Thomas Jefferson University and Jefferson Health, Philadelphia, Pennsylvania, USA.

## Guest Editorial by Stephen K. Klasko, MD, MBA

## Building Ethical and Sustainable Business Models for the Use of AI and Machine Learning in Health Care: Protecting the Human in the Middle

Artificial intelligence (AI) is evolving at warp speed, and new applications enter the market daily. So much attention is fixed on the promise of robotics and machine learning that it's easy to lose sight of the impact of new technologies on the people they're intended to serve. The potential harms and benefits can be amplified when AI is deployed in health care, and unintended consequences can be life-altering.

For generations, science fiction writers have explored scenarios in which machines and humans interact and battle with one another while the fate of the world hangs in the balance. But the books have not yet been written about the kinds of advances we will soon see in machine thinking.

Consider the flurry of activity occurring daily across health care's academic, clinical, business, and entrepreneurial sectors: attention-based learning, neural networks, online-meets-offline (OMO), the Internet of Things (IOT), and the infamous “black box” where the machine writes its own rules of engagement. How much consideration has been given to the human in the middle of these myriad interactions occurring online, offline, and in the cloud?

The purpose of AI in health care is to improve the quality and efficiency of care delivered by clinicians, lower the cost of that care, and provide support for clinical and shared decision making. How much consideration is given to the effect on the humans who program, depend on, and query the technology? What is the impact of information sharing between “Alexa” and Bluetooth-enabled devices (FitBit, continuous glucose monitor, or other wearable) that communicate with physicians, the health system, and public payer electronic health records? How much data should be shared? With whom? For what purpose? And for how long?

Ethicist Aimee van Wynsberghe argues that the AI journey must begin by initiating a meaningful conversation regarding ethics that encompasses values. Responsible use of AI must begin by establishing a climate of trust between the people developing the technology and the people who will be using or exposed to it. A product should be practical, useful, and “trustworthy” before it ever leaves the drawing board.

Computers will always be superior to humans when it comes to storing and quickly processing information, but can a machine ever “learn” self-awareness, cultural sensitivity, and empathy – the uniquely human wisdom gained through life experience? Humans have extraordinary abilities when it comes to anticipating the unexpected and considering health-related options in ways that align with their personal values.

How can we foster a collaborative culture and framework that combines the unique qualities of technology and humans for achieving better population health?

We spent decades learning to mentor students in interprofessional education. The next logical progression is a vehicle to mediate the interactions between humans and AI: a “Center for Inter-Sentient Education” that offers health care professionals a thorough grounding in medical informatics, robotics, and the ethical use of technology.

The “human in the middle” is the right frame for these discussions. Design should focus on the needs and desires of individuals/patients rather than institutions/corporations to ensure these technologies do not just benefit the rich. It all comes down to what kind of society we want for our children and grandchildren.

## THE HUMAN IN THE MIDDLE: PRESENTATION AND REACTOR PANEL PROCEEDINGS

Ideally, AI technology should facilitate the collaborative output of robots and humans to build a future of “health assurance,” whereby people are supported in their ultimate goal of wellness. On December 10, 2019, Thomas Jefferson University in Philadelphia, PA initiated an informative and enlightening dialogue on this important topic.

**Moderator:**

***Stephen K. Klasko, MD, MBA,*** President of Thomas Jefferson University and Chief Executive Officer of Jefferson Health. A Distinguished Fellow of the World Economic Forum, Dr. Klasko is an advocate for transformation of health care delivery and medical education.

**Presenter:**

***Aimee van Wynsberghe, PhD,*** Assistant Professor of Robotics and Ethics, Delft University of Technology in the Netherlands. Dr. Van Wynsberghe, one of Europe's foremost robotics ethicists, presented the ethics standards for AI for the European Commission as part of the High-Level Expert Group on AI. In 2020, she will present those standards to the European Parliament.

**Panelists:**

***Osagie Imasogie, LLM,*** Co-Founder and Senior Managing Partner, PIPV Capital. An acknowledged “serial entrepreneur,” Imasogie has more than 30 years of experience in the fields of law, finance and business management, health care, and the pharmaceutical industry.

***Bon Ku, MD, MPP,*** Assistant Dean for Health and Design, Thomas Jefferson University. A practicing emergency medicine physician, Dr. Ku directs the Health Design Lab and created the first design thinking program at a US medical college.

***Derek O'Halloran, MPA,*** Head of the World Economic Forum's initiative on the future of the digital economy and society.

***Amanda Walker,*** third-year medical student, Sidney Kimmel Medical College. Walker instructs a humanities course titled “Frontiers of Medical Ethics” for first-year medical students.

## Presentation: A Medical Ethics Perspective on Artificial Intelligence in Health Care

Ethicist Aimee van Wynsberghe made a persuasive argument that the journey must begin by initiating a meaningful conversation regarding AI and ethics in the context of health care – a conversation that encompasses values such as data privacy, ownership and governance, access and equity, virtual versus in-person therapy. Developing and using AI technology in a responsible manner requires careful consideration of downstream consequences.

Embedding ethics early in the design process assures that a thorough assessment will be conducted regarding how a product will be used, by whom, and under what conditions. This process leads to better understanding of its value to society and to consumers.

As applied in health care today, use of AI is essentially a real-world experiment conducted without the consent of the individuals it affects. When a product involves health and well-being, there must be explicit acknowledgement of the experimental nature, and simply stated terms and conditions that describe how personal data will be collected, used, and disclosed. There are many questions that require careful consideration, including potential use of data for a purpose unrelated to the original experiment, expected life cycle of the data collected, and inherent bias in algorithms.

As we work to meet growing demands from health care professionals and consumers, we must find efficient ways to expand AI advances to meet the needs of historically underserved populations in addition to those already acculturated to technology. The stakes are high in health care; a medical ethicist on the design team can help uncover, understand, mediate and mitigate risks.

Dr. Van Wynsberghe characterized ethics as a call to action, requiring us to assess our values and evaluate how we ensure, protect, and aid the people in the middle. This is especially true when we develop new products. She believes ethics is integral to the product design process from its inception; in essence, the heart of an ethical business model for AI in health care.

“Ethics is the study of ‘the good life.’ We can define it and create a path to achieving it; but it is an ongoing process, not a checklist. Starting with ethics, we need to envision ’the good life’ we want to achieve. What does that ’good life’ look like, and how do we use AI to help us get there?” – Aimee van Wynsberghe

## Panelist Reactions

### Health Care Industry Business Perspective

Panelist Osagie Imasogie reasoned that meaningful conversation about ethics requires a mutually accepted definition of the term. What one considers as ethical depends on the social and cultural context. AI programming should not begin without taking into account the embedded logic tree that will drive outputs.

We can learn from science fiction. Ironically, it addresses the nuances of ethics and ethical dilemmas in a more thoughtful way than actual science.

### Health Economics Perspective

Panelist Derek O'Halloran of the World Economic Forum argued that even the way data are collected becomes an evaluation of our societal values. The cloud of data and intelligence that AI is gathering can end up one of 2 ways. It could help us design economic and societal systems that are truly human-centric, or it could be used to create wealth for a few, increasing exclusion and inequity.

Conversations about AI are not exclusive to health care, but are happening in many sectors of the consumer space and industry.

On a very basic level, we shape the future through small decisions we make every day. At scale, the process becomes one of adoption and acceptance. We must carefully consider what we want in both economic and societal terms, embedding ethics in all discussions.

The study of ethics in health care often focuses on protecting the patient's rights. The “human in the middle” is each individual in the middle of myriad things that happen in their lives.

### Medical Student Perspective

Panelist Amanda Walker views AI as a powerful tool when complemented by conversation and joint decision making with health care professionals.

*″We have the technology to allow a 70-year-old person to give birth to a child. Should we encourage this? Is that the best option for the child or for society? These are questions that we need to consider.″* – *Amanda Walker*

Thomas Jefferson University's Sidney Kimmel Medical College pioneered a medical education track that focuses on the humanities in terms of what we should do with all the information we have. Medical ethics, a required course for medical students, helps students think through these “what if” scenarios.

### Computer Science and Technology Design Perspective

As an increasing number of consumers make health care decisions based on information that is available and easily accessible online, clinicians must be prepared to have conversations about that information with their patients.

Panelist Bon Ku believes that design thinking begins with empathy (ie, understanding the person who will use the tool) and that successful product development incorporates an “intimate feedback loop” with that user. Regardless of how exciting the technologies might be, programmers should resist the temptation to work in isolation and always include clinicians, patients, and caregivers in product designs.

The electronic health record (EHR), a major AI development in health care, has not lived up to its promise, in large part because of a design flaw. EHR systems are costly to implement and add a complexity burden to the human workload. Many clinicians view the EHR as a barrier to empathy with their patients.

“When I encounter something I like or dislike when working with an electronic health record in the emergency room, I'd appreciate having an intimate feedback loop that allows a developer to make a change in real time.” – Bon Ku

### Perspectives on Privacy

Moderator Stephen Klasko noted that, in the United States, views on privacy tend to vary generationally and socioeconomically. Trust is a huge factor. For example, when free genomic testing was offered system-wide to all Jefferson employees, willing acceptance was concentrated among employees at the lower end of the workforce pay scale. These employees appreciated having access to information about their health risk, whereas employees at the higher end were more concerned about how the data would be used.

According to Dr. Van Wynsberghe, privacy is no longer a big concern in Europe. The General Data Protection Regulation mandates that companies secure explicit permission for tasks requiring data collection, and that they collect the minimum amount of data for completing the task.

Mr. O'Halloran commented that large companies have found ways to combine and share data and drive insights without infringing on privacy rights. Many are doing this via a series of rights and permissions regarding data pools rather than seeking data ownership. He also observed that the most successful start-ups and apps are those that are the most privacy conscious.

Mr. Imasogie added that most people are not thinking carefully about volunteering their personal data because they focus on and have grown accustomed to the benefit they derive from sharing it. Facebook is a prime example – 2.5 billion people who choose to use the app provide a huge amount of data.

“Why would someone give you a free service? Because they will get the data. That is the deal. The person is the product…and that is the ‘quid pro quo’.” – Osagie Imasogie

### Technology and Disparities

Dr. Klasko observed that technology has the potential to increase or decrease disparities depending upon how we use it. He asked panelists to consider policies that might help the industry move toward a greater social good.

Dr. van Wynsberghe referred to the United Nations' sustainable development goals, several of which incorporate AI. In addition to creating awareness and a shared environment to support collaboration, we must consider funding instruments.

Ms. Walker pointed out the increasing importance of data science training for medical students, who need to understand the black box inputs (eg, whether the data are biased) and how to evaluate data output.

According to Mr. O'Halloran, the next waves of innovation will be at the business model level. Businesses face challenges on 2 fronts: their core business models and their roles in society. Successful models will be those that create positive societal outcomes in addition to creating economic value. Interconnectedness will make it possible to serve markets that previously were inaccessible.

Mr. Imasogie believes that intellectual property (IP) is the fundamental issue underlying disparities. “The net worth of 22 human beings now exceeds the net worth of 3.9 billion people (half of the people on the planet) because they had access to and have ownership of IP.” IP is the only asset class that allows unlimited gains in wealth. We must rethink the 20-year protection clause in our patent regulations.

### Implications for Interprofessional Education

Academia has spent decades learning to mentor students in interprofessional education. Dr. Klasko posited that the time has come to support collaborative thinking between humans and machines. An appreciation of ″inter-sentient″ thinking could be revolutionary for academic health centers. In a world of augmented intelligence, future education will be oriented to teaching humans to be humans, not robots.

In 2020, most US medical schools continue to select students based on their science grade point averages, organic chemistry grades, and scores on the standardized Medical College Admissions Test. It should come as no surprise that patients often describe their doctors as lacking empathy, creativity, and good communication skills.

“It's incredibly difficult to deliver an unscheduled, undiagnosed infant with Down syndrome. Inevitably, the parents ask what this means. Good obstetricians explain the chromosomal abnormalities; great obstetricians say, ‘This means you've delivered a beautiful baby who will love you forever. We'll introduce you to other parents who have delivered beautiful babies like yours.’ An artificial brain can't help people understand the meaning of some situations.” – Stephen Klasko

## Conclusion

*The challenge:* To build ethical and sustainable business models for the use of artificial intelligence and machine learning in health care.

*The premise:* Technology can serve as a tool for refocusing US health care delivery from sick care to health assurance (ie, offering both personalized paths to wellness and customized care plans to manage chronic and complex disease). Precision medicine models might be developed to promote population health and reduce health inequities.

A graphic representation of the symposium is available as online Supplemental Data.

## Supplementary Material

Supplemental data

## Figures and Tables

**Figure:**
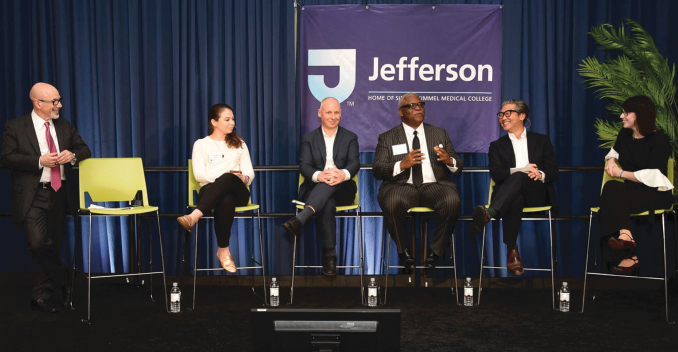
Left to right: Stephen K. Klasko, Amanda Walker, Derek O'Halloran, Osagie Imasogie, Bon Ku, and Aimee van Wynsberghe. Photo courtesy of Thomas Jefferson University. Reproduced with permission.

