# Supplementary material for: The Human in the Middle: Artificial Intelligence in Health Care: Summary Proceedings: Symposium Presentation and Reactor Panel of Experts: Thomas Jefferson University: December 10, 2019
Source: Popul Health Manag. 2021 Apr 9;24(2):282–5. doi: 10.1089/pop.2020.0030 (PMC8060723; doi:10.1089/pop.2020.0030)
Supplement: Supplemental data [file Supp_FigS1.pdf]

Supplementary Data

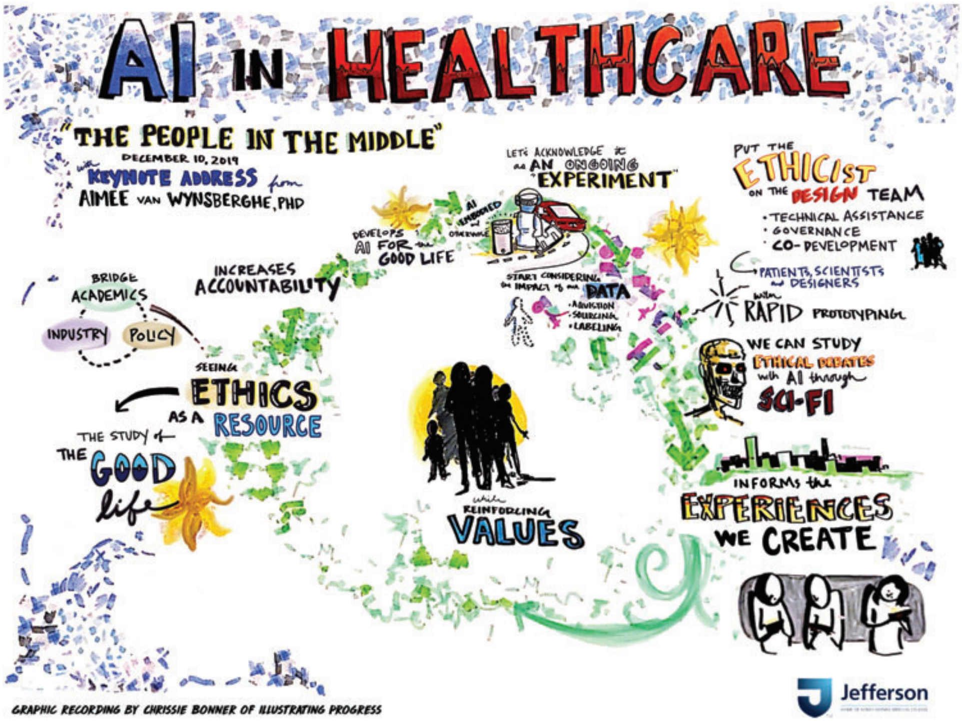

SUPPLEMENTARY FIG. S1. Graphic representation of the proceedings, created during the symposium. Source: Graphic recording by Chrissie Bonner of Illustrating Progress.
